# Phenotypic and Molecular Characterization of Multidrug Resistant *Klebsiella pneumoniae* Isolated from a University Teaching Hospital, China

**DOI:** 10.1371/journal.pone.0095181

**Published:** 2014-04-16

**Authors:** Jikun Du, Peipei Li, Helu Liu, Dongyue Lü, Hong Liang, Yuhong Dou

**Affiliations:** Department of Clinical Laboratory, Shenzhen Shajing Affiliated Hospital of Guangzhou Medical University, Guangzhou, China; St. Petersburg Pasteur Institute, Russian Federation

## Abstract

The multidrug-resistant rate of *Klebsiella pneumoniae* has risen rapidly worldwide. To better understand the multidrug resistance situation and molecular characterization of *Klebsiella pneumoniae*, a total of 153 *Klebsiella pneumoniae* isolates were collected, and drug susceptibility test was performed to detect its susceptibility patterns to 13 kinds of antibiotics. Phenotypic tests for carbapenemases ESBLs and AmpC enzyme-producing strains were performed to detect the resistance phenotype of the isolates. Then PCR amplification and sequencing analysis were performed for the drug resistance determinants. The results showed that 63 strains harbored *bla*_CTX-M_ gene, and 14 strains harbored *bla*_DHA_ gene. Moreover, there were 5 strains carrying *bla*_KPC_ gene, among which 4 strains carried *bla*_CTX-M_, *bla*_DHA_ and *bla*_KPC_ genes, and these 4 strains were also resistant to imipenem. Our data indicated that drug-resistant *Klebsiella pneumoniae* were highly prevalent in the hospital. Thus it is warranted that surveillance of epidemiology of those resistant isolates should be a cause for concern, and appropriate drugs should be chosen.

## Introduction

*Klebsiella pneumoniae* is a common opportunistic pathogen of nosocomial infections that are associated with pneumonia, urinary tract infection, septicemia, as well as bacterial meningitis and biliary tract infection [1], [2]. It can survive in hospitals, persist on environmental surface and colonize human skin, respiratory tract and bowels [3]. Transmission easily occurred among patients via the hands of healthcare personnel [4]. Therefore, *Klebsiella pneumoniae* has become one of the most frequent causes of outbreaks reported in neonatal intensive care units [5].

Antimicrobials have been widely used against *Klebsiella pneumoniae*. However, infections are very refractory to therapeutic interventions. Furthermore, since the overuse of antibiotics and persistent exposure of *Klebsiella pneumoniae* strains to a number of antimicrobials, multidrug-resistant strains have been selected. These strains are resistant to extended-spectrum beta-lactam antibiotics, aminoglycosides, fluoroquinolones, and even the most effective antimicrobial agent of carbapenems [6]. In fact, the increasing isolation of *Klebsiella pneumoniae* non-susceptible to many antibiotics are contributed by some factors, including its innate efflux pump systems to a number of antimicrobial agents, its capacity to acquire resistance determinants and the increased use of antibiotics, which promotes the selection of resistant clones. In particular, these isolates can produce newer beta-lactamases with high hydrolytic activity including extended-spectrum beta-lactamases (ESBLs), AmpC and carbapenem-hydrolyzing enzymes [7]. This problem brings insuperable difficulties in the treatment of infections.

Though many studies have reported the drug resistance of *Klebsiella pneumoniae* worldwide [6]–[8], there is paucity scientific information available on the extent of multidrug-resistant (MDR) *Klebsiella pneumoniae* isolates in Shenzhen. In this study, we aimed to determine the prevalence of MDR strains in our hospital. The samples were collected from the patients in our hospital, and the current situation, phenotypic and molecular characterization of drug resistance *Klebsiella Pneumoniae* isolates were investigated.

## Materials and Methods

### Ethics Statement

All the investigations in our study were approved by the ethics committee of Shenzhen Shajing Hospital affiliated of Guangzhou Medical University, Shenzhen, China. Written consent provided by the patients were stored in the hospital database and used for further research. The medical records of the patients for study were permitted and available from Shenzhen Shajing Hospital. The ethics committee of Shenzhen Shajing Hospital reviewed that relevant ethical issues in this study were all considered.

### Bacterial strains

A total of 153 *Klebsiella pneumoniae* isolates were collected from January 2010 to December 2010 in our hospital. All strains in this study were cultured in Luria-Bertani (LB) medium. Identification of the isolates was performed by the Biomerieux VITEK-2 system. *E.coli* strain ATCC 25922 was used as sensitive control strain, *Klebsiella pneumoniae* strain ATCC 700603 was used as ESBL-producing positive control strain, *Enterobacter cloacae* 029 M was used as AmpC-producing positive control strain, *Klebsiella pneumoniae* strain A1500 was used as carbapenemases- producing control strain.

### Antibiotics

The following antibiotics were provided by the indicated sources: piperacillin/tazobactam and sulbactam/cefoperazone (Toyama Chemical Co., Ltd., Toyama, Japan); cefazolin, cefuroxime, cefotaxime, and cefepime (Farbwerke HoechstAG, Frankfurt, Germany); cefoxitin, ceftazidime, amikacin, gentamicin and levofloxacin (Sigma Chemical Co., St.Louis, Mo); clavulanate (SmithKline Beecham Pharmaceuticals, Surrey, United Kingdom); imipenem and meropenem (Banyu Pharmaceutical Co., Ltd., Tokyo, Japan).

### Drug susceptibility tests

MICs of 13 agents (Sulbactam/Cefoperazone, piperacillin/tazobactam, cefazolin, cefuroxime, ceftazidime, cefotaxime, cefepime, cefoxitin, imipenem, meropenem, amikacin, gentamicin and levofloxacin) were determined by the broth dilution method according to CLSI recommendations [9]. ESBL, AmpC and carbapenemases positive strains should be further conducted by phenotypic test.

### Enzyme extract

A bacterial suspension of 0.5 McFarland was prepared from a single colony of bacterial strain. A volume of 50 µL was subcultured in 12 mL LB broth medium at 37°C for 4 h, then the culture was centrifuged at 4000 g and 4°C for 20 min, the supernatant was discarded and the collecting pellet was frozen and thawed for 5 times. After adding 1.5 mL PBS, the suspension was centrifuged at 9000 g and 4°C for 20 min and finally, the supernatant was stored at −80°C and used as crude enzyme extract.

### Screening of ESBLs-producing strains

Expression of ESBL was detected by ceftazidime or cefotaxime (MIC≥2 µg/mL). Phenotypic test of the ESBLs-producing strains was performed by broth dilution test as described by CLSI guidelines [9]. Briefly, it was performed by testing MICs for cefotaxime and cefotaxime-clavulanic acid, ceftazidime and ceftazidime-clavulanic acid. A threefold concentration decrease in the MIC of either cefotaxime or ceftazidime tested in combination with clavulanic acid comparing with its MIC, ESBLs positive could be considered.

### Screening of AmpC-producing strains

According to the susceptibility results, the positive isolates were further conducted by using three-dimensional test [10].

### Screening of carbapenemases-producing strains

Carbapenemase phenotype was detected by a meropenem or imipenem (MIC≥2 µg/mL). According to the susceptibility results, the positive isolates were further conducted by using modified Hodge test [9].

### Genotype detection

According to the β-lactamase sequences in GenBank database and the documents [11]–[13], the universal primers were designed for β-lactamase genes, including ESBL genes (*bla*_TEM_, *bla*_SHV_ and *bla*_CTX-M_), AmpC genes (*bla*_ACT_, *bla*_DHA_, *bla*_FOX_, *bla*_CMY-G1_ and *bla*_CMY-G2_), carbapenemases genes (*bla*_KPC_, *bla*_GES_, *bla*_IMI/NMC-A_, *bla*_IMP_, *bla*_VIM_, *bla*_GIM_, *bla*_SPM_, *bla*_SIM_, *bla*_NDM-1_ and *bla*_OXA-48_). Details for the primers were shown in Table 1. Sample DNA was extracted by a boiling method that bacteria were heated at 100°C for 10 min in a total volume of 100 µL of ultrapure sterile water followed by centrifugation of the cell suspension. PCR was performed in a 25 µL reaction mixture containing 12.5 µL of GoTaq Green Master Mix (Promega Co., Madison, WI), 0.4 µM of each primer, 3 µL of sample DNA. The PCR conditions used were initial denaturation at 95°C for 5 min, cyclic denaturation at 95°C for 50 s, annealing at 56°C, 50°C or 60°C(details as Table 1) for 40 s, elongation at 72°C for 1 min for 35 cycles and final extension at 72°C for 5 min in a thermocycler (C1000, Bio-Rad Laboratories, Richmond, Calif.), PCR products were detected by 1% agarose gel. Positive amplicons were purified by Promega Wizard SV Gel and PCR Clean-up System (Promega Co., Madison, WI) and sequenced by Beijing Genomics Institute (BGI, China), DNA sequences were annotated by using the BLAST program (http://blast.ncbi.nlm.nih.gov) to identify the gene subtypes.

**Table 1:** Primers used for PCR amplification of resistance genes.

Gene	Primer	Sequence(5′→3′)	Annealing Temp(°C)	Fragment(bp)	Reference
*bla*_TEM_	TEM-F	TCAACATTTCCGTGTCG	56	860	[27]
	TEM-R	CTGACAGTTACCAATGCTTA			
*bla*_SHV_	SHV-F	ATGCGTTATATTCGCCTGTG	56	896	[27]
	SHV-R	AGATAAATCACCACAATGCGC			
*bla*_CTX-M-1_	M-1F	CCGTTTCCGCTATTACAAACCGTTG	56	944	[37]
	M-1R	GGCCCATGGTTAAAAAATCACTGC			
*bla*_CTX-M-2_	M-2F	ATGATGACTCACAGCATTCG	56	833	[38]
	M-2R	TCCCGACGGCTTTCCGCGTT			
*bla*_CTX-M-8_	M-8F	TTTGCCCGTGCGATTGG	50	368	[28]
	M-8R	CGACTTTCTGCCTTCTGCTCT			
*bla*_CTX-M-9_	M-9F	ATGGTGACAAAGAGAGTGCA	50	870	[39]
	M-9R	CCCTTCGGCGATGATTCTC			
*bla*_CTX-M-10_	M-10F	GCAGCACCAGTA AAGTGATGG	56	524	[36]
	M-10R	GCGATATCGTTGGTGGTACC			
*bla*_CTX-M-14_	M-14F	GAGAGTGCAACGGATGATG	56	941	[29]
	M-14R	TGCGGCTGGGTAAAATAG			
*bla*_CMY-G1_	G1-F	GCTGACAGCCTCTTTCTCCAC	56	1082	[40]
	G1-R	CCTCGACACGGRCAGGGTTA			
*bla*_CMY-G2_	G2-F	GGTCTGGCCCATGCAGGTGA	56	963	[40]
	G2-R	GGTCGAGCCGGTCTTGTTGA			
*bla*_DHA_	DHA-F	AACTTTCACAGGTGTGCTGGGT	60	405	[30]
	DHA-R	CCGTACGCATACTGGCTTTGC			
*bla*_ACT_	ACT-F	ATTCGTATGCTGGATCTCGCCACC	50	396	[31]
	ACT-R	CATGACCCAGTTCGCCATATCCTG			
*bla*_FOX_	FOX-F	CACCACGAGAATAACC	50	1184	[31]
	FOX-R	GCCTTGAACTCGACCG			
*bla*_KPC_	KPC-F	TGTCACTGTATCGCCGTCTAG	50	880	[26]
	KPC-R	TTACTGCCCGTTGACGCCCAATCC			
*bla*_GES_	GES-F	ATGCGCTTCATTCACGCAC	56	591	[41]
	GES-R	CTATTTGTCCGTGCTCAGG			
*bla*_IMI_	IMI-F	ATGTCATTAGGTGATATGGC	50	879	[32]
	IMI-R	GCATAATCATTTGCCGTACC			
*bla*_IMP_	IMP-F	GGAATAGAGTGGCTTAATTCTC	50	624	[32]
	IMP-R	CCAAACCACTACGTTATC			
*bla*_VIM_	VIM-F	GATGGTGTTTGGTCGCATA	50	390	[33]
	VIM-R	CGAATGCGCAGCACCAG			
*bla*_GIM_	GIM-F	TCGACACACCTTGGTCTGAA	56	477	[42]
	GIM-R	AACTTCCAACTTTGCCATGC			
*bla*_SPM_	SPM-F	AAATCTGGGTACGCAAACG	56	270	[22]
	SPM-R	AGATTATCGGCTGGAACAGG			
*bla*_SIM_	SIM-F	TACAAGGGATTCGGCATCG	56	570	[42]
	SIM-R	TAATGGCCTGTTCCCATGTG			
*bla*_NDM-1_	NDM-F	TGCCCAATATTATGCACCCGG	60	621	[25]
	NDM-R	CGAAACCCGGCATGTCGAGA			
*bla*_OXA-48_	48-F	TTGGTGGCATCGATTATCGG	56	743	[34]
	48-R	GAGCACTTCTTTTGTGATGGC			

### Conjugation experiment

ESBLs positive isolates were used as donor strains, and streptomycin-resistant *E. coli* C600 was recipient strain. Transconjugants were selected from the Mueller Hinton plate containing streptomycin (1 mg/mL) and ceftazidime (1 µg/mL), and identified with API 20E systems.

## Results

### Drug Susceptibility test

Drug susceptibility test was conducted for 153 isolates of *Klebsiella pneumoniae* and details of resistance to tested drugs were shown in Table 2. Our study indicated that the drug resistance rates of the 153 isolates to cefazolin, cefuroxime, ceftazidime, cefotaxime and cefepime were all high (nearly 40%). In case of aminoglycosides, the non-sensitivity rate of amikacin (13.8%) was lower than that of gentamicin (27.4%). The lowest resistance rates were observed for two carbapenems tested including imipenem (3.3%) and meropenem (2.6%).

**Table 2:** Antimicrobial susceptibilities of 153 strains of *Klebsiella pneumoniae*.

Antibiotics^a^	Range(µg/mL)	MIC_50_(µg/mL)	MIC_90_(µg/mL)	bR%	bI%	bS%	ESBLs-gene positive (*n* = 63)	ESBLs-gene negative (*n* = 90)	AmpC-gene positive (*n* = 14)	AmpC-gene negative (*n* = 139)	KPC-positive (*n* = 5)	KPC-negative (*n* = 148)
							R (%)	R (%)	R (%)	R (%)	R (%)	R (%)
CSL	≦4- ≧32	4	32	7.2	11.1	81.7	9 (14.3)	2 (2.2)	7 (50)	4 (2.9)	4 (80)	7 (4.7)
TZP	≦2- ≧128	8	128	13.7	4.6	81.7	18 (28.5)	3 (3.3)	8 (57.1)	13 (9.4)	4 (80)	17 (11.5)
CZO	≦8- ≧32	8	32	42.5	5.9	51.6	61 (96.8)	4 (4.4)	12 (85.7)	53 (38.1)	5 (100)	60 (40.5)
CXM	≦4- ≧32	4	32	43.1	0.7	56.2	61 (96.8)	5 (5.5)	12 (85.7)	54 (38.8)	5 (100)	61 (41.2)
CAZ	≦4- ≧32	8	32	40.5	0.7	58.8	61 (96.8)	1 (1.1)	10 (71.4)	52 (36.9)	5 (100)	57 (38.5)
CTX	≦4- ≧64	4	32	40.5	1.3	58.2	61 (96.8)	1 (1.1)	10 (71.4)	52 (36.9)	5 (100)	57 (38.5)
FEP	≦4- ≧32	4	32	40.5	0	59.5	61 (96.8)	1 (1.1)	10 (71.4)	52 (36.9)	5 (100)	57 (38.5)
FOX	≦2- ≧32	4	8	7.8	0	92.2	10 (15.9)	2 (2.2)	12 (85.7)	0	5 (100)	7 (4.7)
IPM	≦4- ≧16	4	4	3.3	0	96.7	5 (7.9)	0	5 (35.7)	0	5 (100)	0
MEM	≦2- ≧16	2	2	2.6	0	97.4	4 (6.3)	0	4 (28.6)	0	4 (80)	0
AMK	≦2- ≧64	2	64	13.1	0.7	86.3	14 (22.2)	6 (6.6)	9 (64.3)	11 (7.8)	5 (100)	15 (10.1)
GEN	≦0.5- ≧32	0.5	16	26.1	1.3	72.5	33 (52.4)	7 (7.7)	12 (85.7)	28 (19.9)	5 (100)	35 (23.6)
LVX	≦1- ≧8	1	4	2.6	12.4	85	3 (4.8)	1 (1.1)	3 (21.4)	1 (0.7)	3 (60)	1 (0.7)

a Antibiotics: CSL, Sulbactam/Cefoperazone; TZP, Piperacillin/Tazobactam; CZO, Cefazolin; CXM, Cefuroxime; CAZ, Ceftazidime; CTX, Cefotaxime; FEP, Cefepime;

FOX, Cefoxitin; IPM, Imipenem; MEM, Meropenem; AMK, Amikacin; GEN, Gentamicin; LVX, Levofloxacin.

b R  =  resistance rate,I  =  intermediary rate,S  =  sensitivity rate.

### Detection of ESBLs producing and AmpC producing strains

Among the 153 *Klebsiella pneumoniae* isolates, 51 strains produced ESBLs only, 9 strains produced both ESBLs and AmpC enzymes, and 3 strains only produced AmpC enzyme. Thus, the positive rate of ESBLs-producing strains and AmpC-producing strains was 39.2% and 7.8%, respectively. Sixty ESBLs-producing strains showed multidrug resistance, the proportion of multidrug resistance for ESBL positive strains was higher than that for ESBL negative strains (*P*<0.01). AmpC enzyme producing isolates were resistant to most cephalosporin antibiotics.

### Detection of carbapenemases-producing strains

Of 153 *Klebsiella pneumoniae* isolates, 4 strains were conducted to be carbapenemases positive by using modified Hodge test. All these 4 isolates produced ESBLs and 3 of them produced AmpC enzyme as well. It was shown that the carbapenemases-producing isolates were resistant to most drugs including imipenem and meropenem.

### PCR Amplification and Sequencing

To analyze drug resistance related genes, PCR amplification and sequencing analysis were conducted for 153 *Klebsiella pneumoniae* isolates. ESBL genes including TEM, SHV and CTX-M type were amplified from *Klebsiella pneumoniae*, and two gene types (*bla*_TEM_ and *bla*_CTX-M_) were detected. There were 14 *bla*_TEM_ positive strains and 63 *bla*_CTX-M_ positive strains, respectively. By sequencing analysis, the subtype of *bla*_TEM_ was *bla*_TEM-1_. While *bla*_CTX-M_ contained three subtypes including *bla*_CTX-M-1_ (n = 4), *bla*_CTX-M-9_ (n = 45) and *bla*_CTX-M-14_ (n = 14). The *bla*_DHA_ was detected in 14 AmpC gene positive strains, and sequencing results showed that its subtype was *bla*_DHA-1_. Meanwhile, 5 carbapenemase gene positive strains harboring *bla*_KPC-2_ were observed. The proportion of resistance to most drugs for ESBL positive strains was higher than that for ESBL negative strains. AmpC gene positive isolates were resistant to 13 antimicrobials in different levels, and *bla*_KPC-2_ positive strains were almost resistant to all the antibiotics, as well as the carbapenems. The antimicrobial susceptibilities of beta-lactamase genes positive and negative strains were shown in Table 2. Of these drug resistant isolates, 4 isolated *Klebsiella pneumoniae* harbored three types of genes including *bla*_CTX-M_, *bla*_DHA_ and *bla*_KPC_ together, 12 isolated harbored both *bla*_CTX-M_ and *bla*_DHA_, 5 isolated carried both *bla*_CTX-M_ and *bla*_KPC_ (Table 3).

**Table 3:** Proportion of beta-lactamase antibiotics resistance associated gene detected in *Klebsiella pneumoniae*.

Genes	Strains				
bla_CTX-M_	+^c^	+	+	+	
bla_DHA-1_	+	+			+
bla_KPC-2_	+		+		
n/N^a^ (%)	4/68 (5.9)	8/68 (11.8)	1/68 (1.5)	50/68 (73.5)	2/68 (2.9)
n/N^b^ (%)	4/5 (80)	0	1/5 (20)	0	0

a n/N: No. of designated drug resistance-associated genes/No. of isolates resistant to the corresponding drugs (Antipseudomonal penicillins + beta-lactamase inhibitors, penicillins + beta-lactamase inhibitors, 1st, 2nd, 3rd and 4th generation cephalosporins, Cephamycins).

b n/N: No. of designated drug resistance-associated genes/No. of isolates resistant to the corresponding drugs (Carbapenems).

c +: including the drug resistance-associated gene.

### Conjugation experiment

Among the 63 ESBL positive isolates, successful transconjugation was observed for 21 strains. These transconjugant strains exhibited an ESBL phenotypic profile. Furthermore, PCR analysis and sequencing data confirmed that the 21 transconjugants carried *bla*_CTX-M_, and of these transconjugants, 2 strains carried *bla*_KPC_, the same beta-lactamases as their parental strains.

## Discussion

Due to the wide use of antibiotics, the MDR *Klebsiella pneumoniae* strains isolated are increasing, and even non-sensitive to carbapenems. Current studies mainly focus on a variety of function enzymes produced in *Klebsiella pneumoniae*, including ESBLs, plasmid-mediated AmpCs, and carbapenemases [6]. Global emergence and spread of carbapenemase genes and ESBL genes among *Klebsiella pneumoniae* isolates, poses severe challenges to public health. In this study, we investigated a total of 153 *Klebsiella pneumoniae* isolates from clinical patients, and aimed to evaluate the prevalence and genetic background of drug-resistant *Klebsiella Pneumoniae* strains in our hospital. The proportion of the ESBL positive cases was highest, followed by AmpC-producing stains, and carbapenemases-producing stains. Furthermore, this study indicated that ESBL positivity was closely related to the resistance of most drugs. In recent years, multidrug resistant caused by ESBLs are reported to be associated with higher morbidity and mortality rates [14]. Thus it is warranted that surveillance of epidemiology of those resistant isolates should be concerned.

A total of 153 *Klebsiella pneumoniae* isolates were identified by its effects on the antimicrobials, the highest drug-resistance rate was observed for the third cephalosporin, at more than 40.0%, and the drug-resistance rates to amikacin and gentamicin accounted for 13.1% and 26.1%, respectively. However, those isolates were sensitive to imipenem and meropenem, and the sensitivity rates were no less than 90%. AmpCs-producing-only strains and ESBLs-producing-only strains are highly resistant to the third generation cephalosporins, the former are less sensitive to cephamycin, while ESBLs-producing strains are just the opposite [15]. The commonly used β-lactamase inhibitor including sulbactam and clavulanic acid have a strong inhibitory action on ESBLs, but less inhibition to AmpCs. The drug-resistance rates of ESBLs-producing positive strains to 13 antibiotics are mostly lower than that of China CHINET bacterial drug-resistance surveillance [35]. It is possible that distinct hospitals are monitored. Significant difference of drug-resistance rate was determined (*P*<0.01) between ESBLs positive and negative strains.

The detection of genetic determinants associated with drug resistance to *Klebsiella Pneumoniae* isolates is essential for appropriate antimicrobial therapy and infection control. At present, ESBLs and AmpCs have been predominant β-lactamases that mediate gram-negative bacillus resistance to new broad spectrum β-lactam antibiotics. ESBLs are mainly mediated by plasmid, while AmpCs are mainly mediated by chromosome. Our conjugation experiment had found the transferable ESBL gene. CTX-M types are the major phenotypes of domestic ESBLs, which have been reported to be prevalent in the world [16], followed by SHV type [17]. In this study, among the ESBLs-producing *Klebsiella pneumoniae* isolates, the majority of ESBL genotype was *bla*_CTX-M_, 45 isolates were the subtype of *bla*_CTX-M-9_, 14 isolates were *bla*_CTX-M-14_ and 4 strains were *bla*_CTX-M-1_. All of 14 TEM-genotype *Klebsiella pneumoniae* isolates were *bla*_TEM-1_, but the genotype did not belong to ESBL gene. SHV-type ESBL gene was undetected in our study, its rate was extremely lower than that reported by the previous studies. Veras et al reported that 55.8% of the *Klebsiella pneumoniae* isolates harbored the *bla*_SHV_ genes in Recife, Brazil [18]. Indeed, the prevalence of ESBL genes could vary geographically and time wise. AmpC genotype is given priority to *bla*_CMY_ in the worldwide, especially the subtype of *bla*_CMY-G2_. However, there are paucity studies on AmpC genotypes in China. Chen et al [12] firstly reported the clinical isolated *E.coli* strain that produced AmpC enzyme of *bla*_ACT-1_ genotype. In this study, we had detected 14 isolates carrying *bla*_DHA-1_ gene with PCR method. Indeed, the *Klebsiella pneumoniae* isolates that produce DHA-1 type AmpC are prevalent in Taiwan [19]. Of these 14 isolates, there were *bla*_CTX-M_ coexisting in 12 clinal isolates.

Production of carbapenemases is an important mechanism for *Klebsiella pneumoniae* resistance to carbapenems [20]. These enzymes can hydrolyze not only carbapenems but also most antimicrobial agents. So far, there have been about 70 kinds of carbapenemases reported in the world. The first carbapenemase found in *Klebsiella pneumoniae* was KPC [21], which has been widely reported in the world. At present, KPC-2 is a dominant type of carbapenemase in domestic report, and spread through many cities like Shanghai, Guangzhou and Zhengzhou after its first identification in Zhejiang province [15]. VIM and IMP metallo-beta-lactamase genes are reported with a higher prevalence in southern Europe and Asia [23], [24], but the genes were not found in our study. The results obtained by PCR method indicated that *bla*_KPC-2_ was the unique carbapenemase gene detected in our study, and 5 isolated *Klebsiella pneumoniae* harbored both *bla*_CTX-M_ and *bla*_KPC_ genes, meanwhile *bla*_DHA_ gene was coexistent in 4 of them. It was also shown that MDR *Klebsiella pneumoniae* may be associated with several β-lactamases. Though some recently emerging beta-lactamases such as NDM-1 was undetectable in our study, they should be still concerned and continuous monitoring [25], [26].

The study has revealed that ESBLs positive *Klebsiella pneumoniae* were resistant to the majority of new broad spectrum β-lactam antibiotics, and some strains also carry AmpC and carbapenemase genes together, which lead to multidrug resistance. Particularly, some β-lactamases are mediated by plasmids or transferable gene, and these β-lactamase genes are quite easy to spread to other unrelated clones or to other species, so the isolation of resistant strains is an alarm to establish strict infection control measures preventing the spread of β-lactamase genes. Consequently, the monitoring of drug-resistant isolates and rational use of antimicrobials become significant to limit the spread and prevalence of the underlying resistance mechanisms.
